# Elranatamab in relapsed/refractory multiple myeloma patients on hemodialysis: A real-world case series

**DOI:** 10.46989/001c.171328

**Published:** 2026-09-28

**Authors:** Hikmettullah Batgi, Fatoş Dilan Köseoğlu, Denis Çetin, Semih Başcı, Sinem Namdaroğlu, Nur Akad Soyer

**Affiliations:** 1 Dokuz Eylul University, Faculty of Medicine, Department of Hematology and Bone Marrow Transplantation Unit, Department of Hematology, Izmir, Turkey; 2 Department of Hematology, Faculty of Medicine, Bakırçay University, Izmir, Turkey; 3 Department of Hematology, Ege University School of Medicine, Izmir, Turkey

**Keywords:** Relapsed/Refractory Multiple Myeloma, Elranatamab, Chronic Kidney Disease, Cytokine release syndrome (CRS), Immune effector cell-associted neurotoxicity syndrome (ICANS)

## Abstract

**Background:**

Renal impairment is a frequent and adverse prognostic factor in multiple myeloma (MM), with dialysis-dependent patients largely excluded from pivotal trials of B-cell maturation antigen (BCMA)-directed therapies. Elranatamab, a BCMA×CD3 bispecific antibody, has demonstrated significant efficacy in relapsed/refractory multiple myeloma (RRMM); however, data in end-stage renal disease are extremely limited. We report real-world outcomes of four dialysis-dependent RRMM patients treated with elranatamab.

**Results:**

Patients with a median age of 67 years (range 61–80) had received a median of six prior lines of therapy (range 4–7). All were refractory to proteasome inhibitors, immunomodulatory drugs, and anti-CD38 therapy, and none had prior BCMA-directed treatment. Baseline glomerular filtration rate ranged from 5 to 20 mL/min, and all patients were dialysis-dependent. At data cutoff, all four patients were evaluable for response. Two patients achieved complete response (CR), one achieved very good partial response, and one achieved partial response, resulting in an overall response rate of 100%. Treatment was ongoing in all four patients. Cytokine release syndrome (CRS) occurred in one patient (grade 1) and was self-limited. No immune effector cell-associated neurotoxicity syndrome (ICANS) events were observed. One patient developed severe pneumonia requiring intensive care unit admission; treatment was temporarily interrupted and successfully resumed without step-up dosing, maintaining CR. Hematologic toxicity was limited, with grade-4 lymphopenia in one patient. No dialysis-related complications or unexpected toxicities were observed. All patients received infection prophylaxis after hemodialysis, including trimethoprim–sulfamethoxazole, valacyclovir, and levofloxacin, with intravenous immunoglobulin administered.

**Conclusion:**

In this real-world case series of dialysis-dependent RRMM patients, elranatamab demonstrated high response rates, low incidence of CRS, absence of neurotoxicity, and manageable infectious complications. These findings support the feasibility and clinical activity of elranatamab in patients undergoing hemodialysis. Larger prospective studies are warranted to confirm these encouraging results.

## Introduction

Renal impairment is a common and clinically significant complication of multiple myeloma (MM), affecting approximately 20–40% of patients during the disease course, with a subset progressing to dialysis-dependent renal failure.^1^ The presence of advanced chronic kidney disease (CKD) is associated with inferior survival outcomes and limited therapeutic options.^2^

In the relapsed/refractory multiple myeloma (RRMM) setting, patients who are refractory to proteasome inhibitors (PIs), immunomodulatory drugs (IMiDs), and anti-CD38 monoclonal antibodies have particularly poor prognoses.^3^ B-cell maturation antigen (BCMA)-targeted therapies, including antibody-drug conjugates, CAR-T cells, and bispecific antibodies, have significantly improved outcomes in this population.^4^

Elranatamab is a humanized bispecific antibody targeting BCMA on plasma cells and CD3 on T cells, redirecting cytotoxic T-cell activity against myeloma cells.^5^ In the MagnetisMM-3 trial, elranatamab demonstrated an overall response rate (ORR) of approximately 61% in heavily pretreated RRMM patients, with manageable toxicity.^6^ However, patients with severe renal impairment or those requiring hemodialysis were largely excluded from pivotal studies.

Given the lack of prospective data in dialysis-dependent patients, real-world evidence is critical. Here, we report our experience with four RRMM patients on chronic hemodialysis treated with elranatamab.

## Methods

We conducted a retrospective observational analysis of four consecutive triple-class–refractory RRMM patients with stage 4–5 chronic kidney disease receiving maintenance hemodialysis who were treated with elranatamab between April 2025 and June 2026. Treatment was administered in accordance with the approved Summary of Product Characteristics (SmPC). All patients received the standard step-up dosing regimen followed by weekly elranatamab administration. Those who achieved a sustained response were subsequently switched to every-other-week dosing according to the SmPC recommendations. Responses were assessed according to International Myeloma Working Group (IMWG) criteria. Adverse events were graded per CTCAE v5.0, and cytokine release syndrome (CRS)/immune effector cell-associated neurotoxicity syndrome (ICANS) were graded per ASTCT criteria. Written informed consent was obtained from all individual participants included in the study for publication of clinical data.

## Case 1

A 62-year-old man with IgG lambda MM, diagnosed in 2014 (International Staging System (ISS) stage III), had received six prior lines of therapy, including two autologous stem cell transplantations (ASCT). He was triple-class refractory, having failed PI and IMiD, and anti-CD38 monoclonal antibody therapy, with no prior exposure to BCMA-targeted treatments.

At the initiation of elranatamab on April 2025, the patient had end-stage renal disease (ESRD) with an estimated glomerular filtration rate (eGFR) of 9 mL/min, and was receiving maintenance hemodialysis. Elranatamab was administered according to the approved step-up dosing schedule. No CRS or ICANS were observed.

During treatment, the patient developed severe pneumonia requiring hospitalization and intensive care unit admission, resulting in omission of cycles 8 and 9 and a treatment interruption of approximately two months. The infectious episode resolved with appropriate antimicrobial therapy, and as the interruption was shorter than 12 weeks, elranatamab was resumed without repeating the step-up dosing regimen, in accordance with prescribing recommendations.

At the data cutoff date (June 16, 2026), he had received a total of 14 treatment cycles and remained on active elranatamab therapy with sustained complete response (CR) and no evidence of disease progression. Given the increased risk of infections associated with BCMA-directed therapy, intravenous immunoglobulin (IVIG) replacement together with antiviral, antibacterial, and Pneumocystis jirovecii prophylaxis were continued throughout follow-up.

## Case 2

A 73-year-old female with lambda light chain MM, diagnosed in 2022 (ISS stage III, standard-risk cytogenetics), had received seven prior lines of therapy. She was refractory to PI and IMiD, and daratumumab, had no prior exposure to BCMA-directed therapies, and had not undergone ASCT.

At elranatamab initiation on July 2025, her eGFR was 20 mL/min, and she was receiving maintenance hemodialysis. Treatment was administered according to the standard dosing schedule. The patient experienced grade 1 CRS, which resolved spontaneously without specific intervention. No ICANS or severe infectious complications were observed during follow-up.

The patient achieved a very good partial response (VGPR) after six cycles of elranatamab. Given the favorable efficacy and tolerability profile, treatment was continued beyond cycle 6. During follow-up, the depth of response further improved, resulting in a CR. At the data cutoff date (June 16, 2026), the patient had received 12 cycles of elranatamab and remained on active treatment with ongoing CR and no evidence of disease progression.

## Case 3

A 61-year-old man was diagnosed with IgA lambda MM in 2015. At diagnosis, he had ISS stage I disease and standard-risk cytogenetics. Before initiation of elranatamab, the patient had received seven prior lines of therapy and was refractory to PI, IMiD, and daratumumab. He had not undergone ASCT and had no prior exposure to BCMA-directed therapies.

The patient developed ESRD secondary to MM and required chronic hemodialysis. At the start of elranatamab treatment on October 2025, his eGFR was 8 mL/min. Elranatamab was administered according to the standard dosing schedule, and antimicrobial prophylaxis consisting of trimethoprim-sulfamethoxazole, valacyclovir, levofloxacin, and IVIG replacement therapy were provided following dialysis sessions.

No CRS, ICANS, or severe infectious complications occurred during treatment. The patient developed grade 4 lymphopenia during treatment, which subsequently resolved. At the data cutoff, the patient had completed nine cycles of elranatamab over 9 months and remained on therapy. The best response achieved was a partial response (PR), which was ongoing at the time of analysis.

## Case 4

An 80-year-old man was diagnosed with lambda light-chain MM in 2020. The disease was classified as ISS stage III with standard-risk cytogenetic features. Prior to elranatamab treatment, he had received four previous lines of therapy and had become refractory to PI, IMiD, and daratumumab. He had not undergone ASCT and did not receive prior BCMA-targeted therapy.

The patient had severe renal impairment requiring maintenance hemodialysis, with an eGFR of 5 mL/min before treatment initiation. Elranatamab therapy was started on January 2026. Prophylactic treatment with trimethoprim-sulfamethoxazole, valacyclovir, levofloxacin, and IVIG was administered throughout treatment.

Elranatamab was well tolerated, with no episodes of CRS, ICANS, or severe infection observed. At the data cutoff, the patient had completed six cycles of therapy corresponding to 6 months of treatment exposure. VGPR was achieved and remained ongoing. The patient was alive and continued receiving elranatamab treatment at the time of analysis.

## Patient Characteristics

The median age was 67 years (range 61–80), and three patients were male. Two patients had light-chain MM, one had IgG lambda, and one had IgA lambda disease. ISS stage was III in three patients and I in one patient.

Patients had received a median of six prior lines of therapy (range 4–7). All were refractory to PIs, IMiDs, and daratumumab. One patient had undergone two prior ASCT. All patients were dialysis-dependent, with pre-treatment eGFR values ranging from 5 to 20 mL/min.

## Treatment Exposure and Response

At data cutoff, four patients were evaluable for response: two achieved CR, 1 VGPR and 1 PR. Treatment was ongoing in all four patients.

## Safety

Elranatamab demonstrated a favorable safety profile in this cohort of four heavily pretreated relapsed/refractory MM patients receiving chronic hemodialysis. All successfully completed the step-up dosing schedule and remained on treatment at the time of data cutoff.

CRS was observed in only one patient (25%), occurring as grade 1 toxicity and resolving with supportive care. No grade ≥2 CRS events were reported. Importantly, no cases of ICANS were observed.

One patient developed pneumonia during treatment, representing the only severe infectious complication in the cohort. Elranatamab therapy was temporarily interrupted during the management of the infection and was subsequently resumed following complete clinical recovery. No opportunistic infections, treatment-related deaths, or infection-related permanent treatment discontinuations occurred.

All patients received antimicrobial prophylaxis consisting of trimethoprim-sulfamethoxazole, valacyclovir, and levofloxacin, together with IVIG replacement therapy administered after hemodialysis sessions.

No unexpected toxicities attributable to severe renal impairment or hemodialysis were identified. Furthermore, no patient required permanent discontinuation of elranatamab because of adverse events. At the time of analysis, all four patients remained alive and continued treatment with ongoing clinical benefit.

## Discussion

Dialysis-dependent MM encompasses a high-risk and underrepresented population in prospective clinical trials. Severe renal impairment is associated with inferior survival outcomes and often limits access to novel immunotherapies.^1,7^ In the pivotal MajesTEC-1 (teclistamab) and MagnetisMM-3 (elranatamab) studies, patients with advanced renal failure were either excluded or markedly underrepresented, and dialysis-dependent patients were not included.^5,8^ Therefore, real-world data are essential to guide therapeutic decisions in this vulnerable subgroup.

To date, only three published reports have described the use of elranatamab in dialysis-dependent RRMM patients^9–11^ (Table 2). Across these cases, treatment was feasible, CRS was limited to Grade 1 events, ICANS was not observed, and deep responses—including CR and MRD negativity—were achieved. Importantly, no unexpected toxicities related to renal failure or hemodialysis were reported. These observations suggested that, despite the absence of prospective pharmacokinetic validation in ESRD, elranatamab may be safely administered in this setting.

**Table 1.** Summary of four of our cases treated with elranatamab.

**Characteristic**	**Case 1**	**Case 2**	**Case 3**	**Case 4**
**Age / Gender**	62 / Male	73 / Female	61 / Male	80 / Male
**Year of Diagnosis**	2014	2022	2015	2020
**Myeloma Subtype**	IgG/Lambda light chain	Lambda light chain	IgA/Lambda light chain	Lambda light chain
**ISS Stage**	3	3	1	3
**Cytogenetics**	Unknown	Standard risk	Standard risk	Standard risk
**Prior Lines of Therapy**	6	7	7	4
**Autologous Transplant**	Twice	None	None	None
**PI/IMiD/Dara Refractory**	Yes	Yes	Yes	Yes
**Prior BCMA Exposure**	None	None	None	None
**GFR (pre-Elra)**	9 ml/min	20 ml/min	8 ml/min	5 ml/min
**Hemodialysis**	Yes	Yes	Yes	Yes
**Elra Start Date**	Apr 2025	Jul 2025	Oct 2025	Jan 2026
**Treatment duration at data cutoff: months**	14	12	9	6
**Number of Elra Cycles**	14 cycles (ongoing)	12 cycles (ongoing)	9 cycles (ongoing)	6 cycles (ongoing)
**Best Response**	CR	CR	PR	VGPR
**CRS**	None	Grade 1	None	None
**ICANS**	None	None	None	None
**Severe Infection**	Pneumonia	None	None	None
**Prophylaxis (after hemodialysis)**	TMP-SMX, Valacyclovir, Levofloxacin, IVIG	TMP-SMX, Valacyclovir, Levofloxacin, IVIG	TMP-SMX, Valacyclovir, Levofloxacin, IVIG	TMP-SMX, Valacyclovir, Levofloxacin, IVIG
**Current Status**	Alive, on elranatamab (CR)	Alive, on elranatamab (CR)	Alive, on elranatamab (PR)	Alive, on elranatamab (VGPR)

ISS; International Staging System, PI; proteasome inhibitor, IMiD; immunomodulatory drug, Dara; Daratumab, BCMA; B-cell maturation antigen, GFR; Glomerular Filtration Rate Elra; Elranatamab, CRS; cytokine release syndrome, ICANS; immune effector cell-associated neurotoxicity syndrome, TMP-SMX; Trimethoprim/sulfamethoxazole, IVIG; Intravenous immunoglobulin.

**Table 2.** A summary of three cases treated with Elranatamab in the literature.

**Characteristic**	**Case 1**	**Case 2**	**Case 3**
**Age / Gender**	79 / Female	72 / Female	68 / Female
**Myeloma Subtype**	IgA/Lambda light chain	IgG/Lambda light chain	IgG/κappa light chain
**R-ISS Stage**	Stage III	Not specified (del17p+)	Stage III
**High-Risk Cytogenetics**	T (4;14)	Del (17p)	Del (17p), gain 1q21, t (11;14), del16q, hypodiploidy
**Year of Diagnosis**	2018	2013	2023
**Renal Status**	Dialysis-dependent (since 2020)	Dialysis-dependent (since 2022)	Dialysis-dependent at diagnosis
**Prior Therapies**	4	Multiple lines + ASCT + IsaPd	1
**Elranatamab Initiation**	August 2023	2022	September 2024
**Administration**	12–32–76 mg step-up, post-HD	12–32–76 mg step-up, post-HD	12–32–76 mg step-up, post-HD
**CRS**	Grade 1 (prolonged; tocilizumab required)	Grade 1 (single tocilizumab dose)	Recurrent Grade 1 (3 doses tocilizumab + steroids)
**ICANS**	None	None	None
**Cytopenias**	None significant	Transient Grade 2 thrombocytopenia	Infection-related transient thrombocytopenia
**Other Complications**	COVID-19 pneumonia	Cholangitis; mild hypotension	Exfoliative dermatitis
**Infection Prophylaxis**	TMP-SMX + valacyclovir	Not specified (Ig replacement given)	TMP-SMX + valacyclovir
**Response**	CR at 1 month	sCR + MRD-negative	CR + MRD-negative (4 months)
**Current Status**	Ongoing CR at 4 months	Ongoing CR at 11 months	Ongoing CR (dialysis-dependent)

R-ISS; Revised International Staging System, ASCT; autologous stem cell transplant, IsaPd; isatuximab, pomalidomide, dexamethasone, post-HD; post hemodialysis, CRS; cytokine release syndrome, ICANS; immune effector cell-associated neurotoxicity syndrome; COVID-19; coronavirus disease 2019, TMP-SMX; Trimethoprim/sulfamethoxazole, sCR; string complete remission, MRD; Minimal Residual Disease.

Our real-world cohort of four dialysis-dependent RRMM patients further expands this limited evidence base. All patients were heavily pretreated and triple-class refractory, and none had prior exposure to BCMA-directed therapy. Despite advanced renal dysfunction (eGFR 5–20 mL/min) and chronic hemodialysis, elranatamab demonstrated substantial clinical activity. Among the four evaluable patients, ORR was 100%, including two CR (50%), one VGPR (25%), and one PR (25%). Notably, 75% of patients achieved a response of at least VGPR. Although limited by the small sample size, these outcomes compare favorably with the MagnetisMM-3 trial, which reported an ORR of 61% and a CR/sCR rate of 31.7% in heavily pretreated patients receiving elranatamab.^5^

Safety findings were similarly reassuring. CRS was uncommon and limited to a single Grade 1 event in our series, consistent with previously reported dialysis cases^9–11^ and with the known CRS profile of anti-BCMA bispecific antibodies.^5,8^ No ICANS was observed, aligning with both prior dialysis case reports and larger clinical trials. These data suggest that ESRD does not appear to increase the risk of neurotoxicity.

Infectious complications remain the principal safety concern with BCMA-targeted bispecific antibodies. Profound hypogammaglobulinemia and sustained T-cell redirection predispose patients to bacterial and viral infections.^12,13^ Clinical studies have reported grade ≥3 infections in approximately 20–30% of patients receiving BCMA bispecific antibodies.^5,8^ In previously published dialysis-dependent elranatamab cases, serious infections—including SARS-CoV-2 pneumonia and upper respiratory tract infections—were reported despite vaccination and prophylaxis.^9–11^ Similarly, in our cohort, one patient developed severe pneumonia requiring intensive care but subsequently recovered and continued therapy. Routine antimicrobial prophylaxis and IVIG replacement likely mitigated infection-related morbidity. These findings reinforce that dialysis itself constitutes an additional infectious risk factor,^14^ necessitating vigilant monitoring and proactive supportive strategies.

Population pharmacokinetic analyses indicate that mild to moderate renal impairment does not significantly alter elranatamab exposure.^5^ Although data in ESRD remain limited, no accumulation-related toxicities were observed in our cohort or prior reports,^9–11^ supporting the biological plausibility of standard dosing in dialysis-dependent patients. Administration after hemodialysis sessions, as performed in most reported cases, represents a pragmatic approach in the absence of formal pharmacokinetic studies.

When contextualized within the broader landscape of BCMA-directed therapies, emerging real-world data with teclistamab in dialysis-dependent patients have demonstrated encouraging response rates without excess high-grade CRS or ICANS, although infections remain frequent.^15,16^ Similarly, retrospective analyses of idecabtagene vicleucel (CAR-T therapy) in patients with renal impairment—including ESRD—have shown comparable efficacy but higher rates of transient cytopenias.^17^ Collectively, these findings suggest that advanced renal dysfunction should not automatically preclude BCMA-targeted immunotherapy, provided that comprehensive supportive care is implemented.

Our study is limited by a small sample size and relatively short follow-up in some patients. Nevertheless, when integrated with the three previously published elranatamab dialysis cases,^9–11^ the cumulative evidence now encompasses seven reported dialysis-dependent patients treated with elranatamab. Across these cases, response rates have been consistently high, CRS has been predominantly low grade, neurotoxicity has not been observed, and infectious complications have been manageable with appropriate prophylaxis.

## Conclusion

In this real-world case series of four dialysis-dependent patients with RRMM, treatment with elranatamab demonstrated encouraging clinical activity and a favorable safety profile. High response rates (CR/VGPR/PR) were achieved, while the incidence of CRS was low and no neurotoxicity was observed. Infectious complications were manageable.

Patients requiring hemodialysis are frequently excluded from prospective clinical trials evaluating novel myeloma therapies, resulting in a significant evidence gap for this vulnerable population. Our findings suggest that elranatamab may be a feasible and effective therapeutic option for RRMM patients undergoing hemodialysis. Larger prospective studies are needed to further confirm the safety and efficacy of elranatamab in this underrepresented patient group.

### Disclosure

The authors have no conflicts of interest.

### Authors’ Contribution- CRediT

Conceptualization: Sinem Namdaroğlu

Data curation: Hikmettullah Batgi, Fatos Dilan Koseoglu, Denis Cetin

Supervision: Nur Akad Soyer

Writing – original draft: Hikmettullah Batgi

Writing – review & editing: Semih Başcı

### Informed Consent Statement

All authors and institutions have confirmed this manuscript for publication.

## Data Availability

All are available upon reasonable request.

## References

[1] Gavriatopoulou M., Terpos E., Kastritis E.. (2016). Current treatments for renal failure due to multiple myeloma. Expert Opin Pharmacother.

[2] Haynes R. J., Read S., Collins G. P.. (2010). Presentation and survival of patients with severe acute kidney injury and multiple myeloma: a 20-year experience from a single centre. Nephrol Dial Transplant.

[3] Gandhi U. H., Cornell R. F., Lakshman A.. (2019). Outcomes of patients with multiple myeloma refractory to CD38 targeted monoclonal antibody therapy. Leukemia.

[4] Kumar S. (2021). Multiple Myeloma: From Baby Steps to Giant Strides. JCO Oncol Pract.

[5] Lesokhin A. M., Tomasson M. H., Arnulf B.. (2023). Elranatamab in relapsed or refractory multiple myeloma: phase 2 MagnetisMM-3 trial results. Nat Med.

[6] Costa L. J., LeBlanc T. W., Tesch H.. (2024). Elranatamab efficacy in MagnetisMM-3 compared with real-world control arms in triple-class refractory multiple myeloma. Future Oncol.

[7] Bladé J., Rosiñol L. (2007). Complications of multiple myeloma. Hematol Oncol Clin North Am.

[8] Moreau P., Garfall A. L., van de Donk N. W. C. J.. (2022). Teclistamab in Relapsed or Refractory Multiple Myeloma. N Engl J Med.

[9] Van de Wyngaert Z., Romera-Martinez I., Chedeville C.. (2024). Elranatamab treatment in a multiple myeloma patient undergoing renal dialysis. Clin Hematol Int.

[10] Hoffmann M., Jeker B., Huynh-Do U.. (2025). Elranatamab for Relapsed/Refractory Multiple Myeloma With Severe Renal Impairment Requiring Hemodialysis. Hematol Oncol.

[11] Ogiya D., Suzuki R., Goto S.. (2025). Efficacy and safety of bispecific antibodies in multiple myeloma patients with end-stage renal disease undergoing hemodialysis: a case report of elranatamab and a literature review. Ann Hematol.

[12] Lancman G., Parsa K., Kotlarz K.. (2023). IVIg Use Associated with Ten-Fold Reduction of Serious Infections in Multiple Myeloma Patients Treated with Anti-BCMA Bispecific Antibodies. Blood Cancer Discov.

[13] Rodriguez-Otero P., Usmani S., Cohen A. D.. (2024). International Myeloma Working Group immunotherapy committee consensus guidelines and recommendations for optimal use of T-cell-engaging bispecific antibodies in multiple myeloma. Lancet Oncol.

[14] Kato S., Chmielewski M., Honda H.. (2008). Aspects of immune dysfunction in end-stage renal disease. Clin J Am Soc Nephrol.

[15] Lachenal Florence, Lebreton Pierre, Bouillie Sylvie. (2023). Teclistamab in Relapsed Refractory Multiple Myeloma Patients on Dialysis: A French Experience. Blood.

[16] Roila Nutdanai, Suvannasankha Attaya, Abonour Rafat (2024). Safety and Efficacy of Teclistamab in a Patient Series with Relapsed/Refractory Multiple Myeloma and Severe Renal Impairment on Dialysis. Blood.

[17] Sidana S., Peres L. C., Hashmi H.. (2024). Idecabtagene vicleucel chimeric antigen receptor T-cell therapy for relapsed/refractory multiple myeloma with renal impairment. Haematologica.

